# Calcium Phosphate Based Three-Dimensional Cold Plotted Bone Scaffolds for Critical Size Bone Defects

**DOI:** 10.1155/2014/852610

**Published:** 2014-02-26

**Authors:** Christian J. D. Bergmann, Jim C. E. Odekerken, Tim J. M. Welting, Franz Jungwirth, Declan Devine, Ludovic Bouré, Stephan Zeiter, Lodewijk W. van Rhijn, Rainer Telle, Horst Fischer, Pieter J. Emans

**Affiliations:** ^1^Department of Dental Materials and Biomaterials Research, RWTH Aachen University Hospital, Pauwelsstrasse 30, 52074 Aachen, Germany; ^2^Department of Orthopaedic Surgery, CAPHRI School for Public Health and Primary Care, Maastricht University Medical Centre, P.O. Box 5800, 6202 AZ Maastricht, The Netherlands; ^3^AO Research Institute Davos, Clavadelerstrasse 8, 7270 Davos Platz, Switzerland; ^4^Department of Ceramics and Refractory Materials, Institute of Mineral Engineering, RWTH Aachen University, Mauerstrasse 5, 52064 Aachen, Germany

## Abstract

Bone substitutes, like calcium phosphate, are implemented more frequently in orthopaedic surgery to reconstruct critical size defects, since autograft often results in donor site morbidity and allograft can transmit diseases. A novel bone cement, based on **β**-tricalcium phosphate, polyethylene glycol, and trisodium citrate, was developed to allow the rapid manufacturing of scaffolds, by extrusion freeform fabrication, at room temperature. The cement composition exhibits good resorption properties and serves as a basis for customised (e.g., drug or growth factor loaded) scaffolds for critical size bone defects. *In vitro* toxicity tests confirmed proliferation and differentiation of ATDC5 cells in scaffold-conditioned culture medium. Implantation of scaffolds in the iliac wing of sheep showed bone remodelling throughout the defects, outperforming the empty defects on both mineral volume and density present in the defect after 12 weeks. Both scaffolds outperformed the autograft filled defects on mineral density, while the mineral volume present in the scaffold treated defects was at least equal to the mineral volume present in the autograft treated defects. We conclude that the formulated bone cement composition is suitable for scaffold production at room temperature and that the established scaffold material can serve as a basis for future bone substitutes to enhance *de novo* bone formation in critical size defects.

## 1. Introduction

Synthetic, bioresorbable materials are widely used for the treatment of bone defects. Depending on the operation, the usage of autologeous bone is not always the best treatment, for example, in case of a tumour resection or difficult conditions at a possible donor site, like the iliac wing. Therefore the development of scaffolds consisting of synthetic materials is of great interest within the implantology research. The chemistry of the material is an important factor that influences the response of the surrounding tissue. The chemistry of a material, including element and phase composition, can determine if an implant is bioactive, bioresorbable, or bioinert [1]. Another important factor is the porosity of an implant. Preclinical studies show that porous scaffolds with good pore interconnectivity, implanted in large defects, achieve proper vascularisation and enhance the remodelling process [2]. The combination of micro- and macroporosity influences the osteointegration of an implant and determines the formation of new mineralized bone [3]. In particular, the osteointegration and vascularisation of open porous scaffolds are influenced in the first weeks by their microporosity [4].

There are several ways to generate scaffolds using rapid manufacturing methods, like powder bed based 3D printing, selective laser melting, and inkjet printing [5, 6]. These different methods have their advantages and disadvantages regarding resolution, processable materials, and mechanical strength of the manufactured scaffolds [7]. Usually a heating process of often several hundred degrees Celsius is part of the fabrication process, either during the manufacturing or during a subsequent sintering step [8, 9]. This heating step does not allow the incorporation of bioactive materials like BMP's, antibiotics, or microsphere carriers into the scaffold [10].

Bone cements in the form of paste can, besides being easily shaped, harden at low temperatures by means of a chemical treatments, for example, a strong acid [11]. A suitable way to form customized scaffolds from a pasty material is extrusion freeform fabrication [12–15]. Thin threads of material are extruded through a fine nozzle and deposited in layers on top of each other. Scaffolds with different pore sizes can be synthesized by this technique [16–18]. The advantage of the extrusion process is that a lot of different materials can be processed and there is no inherent microporosity like in particle binding methods (e.g., 3D printing), which can influence the mechanical properties [7].

The main issue concerning the extrudability of a bone cement is determined by factors like the liquid to powder ratio (LPR), the plastic limit (which is the smallest liquid to powder ratio needed to form plastic paste), the grain size distribution, the zeta-potential, and steric stabilisation [19–21]. The zeta-potential and the steric stabilisation determine the repulsion of the particles within a suspension and therefore influence their distances and relative movement. The synthesis, cement preparation, and the additive system are influencing these factors. In addition to the material composition, also the mixing and extrusion devices are important factors for the extrudability of bone cement. Research studies regarding the injectability of polymethylmethacrylate (PMMA) cements showed that mixing by hand or by using a vacuum mixer influences the creep strain of the cement [22].

Due to the need of suitable and resorbable scaffolds for bone critical size defects, we propose that a bone cement composed out of *β*-tricalcium phosphate (*β*-TCP), PEG, and citric acid is a suitable material for 3D plotting of bone scaffolds at room temperature and that the use of these scaffolds results in limited *in vitro* toxicity and good* in vivo* bone remodelling in a critical size defect animal model. The cement was designed as such that it would allow the incorporation of an arbitrary, secondary component, which would not participate in the cement reaction. For our research, we intended to use PMMA particles, which is a currently used carrier for *in vivo* drug delivery. The stability and bone remodelling supported by such constructs can only be evaluated in an *in vivo* bone model; therefore cold plotted scaffolds were implanted in the iliac wing of sheep [23] after initial *in vitro* cytocompatibility screening. Histological examination and quantitative microCT imaging of the defect region were used to assess scaffold resorption and bone remodelling.

## 2. Materials and Methods

### 2.1. Synthesis of the Cement Components

The calcium to phosphor ratio of calcium deficient hydroxyapatite (Merk, Darmstadt, Germany) was adjusted to 1.5 by adding 5 wt.-% of dicalcium phosphate dihydrate (DCPD, Merk, Darmstadt, Germany). The components were mixed in a tumble mixer (Turbula, Willy A. Bach, Basel, Switzerland) and heated for one hour at 1000°C to obtain *β*-TCP. The phase purity of the material was verified by XRD (PW 1830, Philips, Eindhoven, Netherlands) analysis.

The synthesized *β*-TCP was milled with distilled water in an agitator ball mill. The suspension was milled for 2 hours with zirconia grinding balls (*Ø* = 1 mm, Tosoh, Tokyo, Japan) at 1500 rpm. The grain size and the specific surface area were determined by laser granulometry (Mastersizer 2000, Malvern, Worcestershire, Great Britain) before and after the milling process. The milled suspension was granulated by spray drying using a spray tower (Mobile Minor, Niro, Soeborg, Denmark) at 235°C inlet temperature and an air pressure of 1.5 bar. The shape of the obtained granulate was examined by scanning electron microscopy (FEI ESEM XL30 FEG, Philips, Eindhoven, The Netherlands).

### 2.2. Cement Formulation and Scaffold Production

The cement used for the production of the scaffolds was composed out of 49 wt.-% *β*-TCP, 23.5 wt.-% PEG 400, 9.8 wt.-% PEG 10000, 5.9 wt.-% citric acid solution, and 11.8 wt.-% distilled water. Cement cylinders for *in vitro* evaluation were produced in cylindric plastic moulds (6.5 mm in diameter and 11 mm in height) at room temperature and were subsequently, after drying, hardened by infiltration with 3 M phosphoric acid for ten minutes and rinsing with demineralised water afterwards.

Plotting of the initial scaffolds for *in vivo* evaluation was performed by a computer controlled linear XYZ-stage. The cement was mixed before the initial plotting process by using a 0.5 mm nozzle with 1.5 mm between plotted strands in the same plane and a subsequent rotation of 60 degrees between layers to eventually establish 6, 16 mm wide and 10 mm high, cylindrical scaffolds without and 6 equally sized scaffolds with 10 wt.-% PMMA granules with a granule size of 20 *μ*m (Dolphys Medical, Eindhoven, The Netherlands), added during the mixing process of the cement. Plotted scaffolds were subsequently hardened as described above. Scaffolds used for *in vivo* evaluation were sterilized by gamma-irradiation (Isotron, Ede, The Netherlands).

### 2.3. *In Vitro* Cell Cytocompatibility Tests

Since the majority of critical size bone defects are being remodelled through endochondral ossification [24], the murine endochondral cell line ATDC5 (RBC0565, Riken BRC, Japan) was used to assess the effect of the *β*-TCP bone cement on cell viability. ATDC5 cells were cultured in medium which was either unconditioned or preconditioned with the above described ceramic cylinders. *In vitro* evaluations were performed in accordance with ISO 10993-5.

Proliferation medium consisted out of DMEM/F12 (Invitrogen, Carlsbad, CA, USA) with 5% FCS (PAA, Pasching, Austria), 1% antibiotic/antimycotic (Invitrogen), and 1% NEAA (Invitrogen). Differentiation medium consisted of proliferation medium supplemented with 10 *μ*g/mL insulin (human, Sigma, St. Louis, USA), 10 *μ*g/mL transferrin (human, Roche Applied Science, Penzberg, Germany) and 30 nM sodium selenite (Sigma). Medium preconditioning was performed by incubating 1 cylinder in 10 mL of culture medium for 24 hours at culture conditions (humidified atmosphere at 37°C and 5% CO_2_), prior to the initial use in the experiment. The unconditioned proliferation/differentiation medium was used as a control.

ATDC5 cells were cultured in 6-well plates at 6400 cells/cm^2^ and were allowed to adhere overnight. Culture media were changed the following day to either unconditioned or preconditioned proliferation medium, which was refreshed every 2 days. ATDC5 proliferation was assessed at day 0 till day 5 and differentiation at day 0 till day 14 (day 0 being the day when normal culture medium was replaced by preconditioned culture medium). Crystal violet staining was used to assess cell proliferation by DNA content. The extracted amount of crystal violet was determined at 590 nm on a spectrophotometer (Bio-Rad, Hercules, CA, USA). Alcian blue staining was used to asses extracellular matrix production. The extracted amount of alcian blue was determined at 645 nm on a spectrophotometer (Bio-Rad, Hercules, CA, USA). The change in absorbance over time was calculated relatively to the average absorption at day 0.

The *in vitro* stability of the plotted scaffold was evaluated by incubating the scaffold in unconditioned proliferation medium under standard culture conditions for 4 weeks.

### 2.4. *In Vivo* Scaffold Evaluation

Twelve female adult Swiss White Alpine sheep were used in this study (skeletally mature, with a weight range of 69–75.5 kg). Six sheep served as a control group, each with a bilateral defect and one filled with autograft (from the complete harvested cylinder from the contralateral side); the defect in the contralateral iliac wing was left empty. The remaining 6 sheep served as the experimental group, where *β*-TCP scaffolds were implanted bilaterally in the iliac wing, a plain *β*-TCP scaffold on one side, and a *β*-TCP scaffold containing PMMA granules in the contralateral iliac wing.

The surgical procedure was performed under general isoflurane (2%) anesthesia, with a diazepam (Valium, 0.3 mg/kg I.V.), ketamine (Ketasol-100, 2 mg/kg I.V.), and propofol (Propofol 1% Fresenius, 2 mg/kg I.V.) as premedication. General anaesthesia was supported by spinal administration of xylazine (Rompun, 0.05 mg/kg) between the sixth and seventh lumbar vertebra. Incision site was sedated by local infiltration with lidocaine (Ultracain). Sheep were positioned in sternal recumbency after which the iliac wings were draped for surgery. A curved incision over the iliac wing was made, to expose the bone. A custom made surgical jig was mounted on the iliac wing, to guide a 17 mm trephine, on the position where the wing was approximately 1 cm thick. Autograft material was grinded on-site and placed in the defects; the extruded scaffolds were press-fit into the defects. The wound was closed in layers and the sheep were allowed to recuperate from the surgical procedure. The first 3 postoperative days' animals received pain medication by daily administration of carprofen (Rimadyl, 4 mg/kg S.C.); after this period, buprenorphine (Temgesic, 0.1 mg/kg I.M.) was administered if needed.

The animals were checked frequently by trained personnel. Wellbeing and behaviour as well as physical and physiological status were closely monitored. After 3 months, the animals were euthanized, by pentobarbital overdose. The defects were drilled out directly post-mortem with a 25 mm core-drill and subsequently fixated in 70% ethanol.

The animal study protocol (GCTM study number CHTOV 0109-ID) and animal experimental procedures were approved by the Graubünden Animal Commission and performed in accordance with the Swiss Animal Protection Law at the AO Foundation Animal facilities in Davos Switzerland by GCTM.

### 2.5. *Ex Vivo* Scaffold Evaluation

Explants were first analysed by microCT imaging (*μ*CT40, Sanco Medical, Switzerland) to determine the volume and the density of the material in the defect. The initial microCT data collection was performed at 70 kVp and 114 *μ*A with a resolution of 36 *μ*m. To determine the bone mineral volume in the tissue by quantitative microCT, a threshold of 283–2781 mg HA/cm^3^ for bone was established by using the empty and autograft samples and the surrounding tissue as a reference. To differentiate between scaffold and bone, the density threshold for an unimplanted *β*-TCP scaffold was determined at 747–2781 mg HA/cm^3^; this value was subsequently used in the assessment of the implanted *β*-TCP scaffolds. Tissue within a threshold of 283–747 mg HA/cm^3^ was considered as remodelled bone. The threshold for *β*-TCP/PMMA scaffolds was determined at 670–2781 mg HA/cm^3^ (due to the PMMA content). For the *β*-TCP/PMMA samples tissue within a threshold of 283–670 mg HA/cm^3^ was considered as remodelled bone.

After microCT analysis, the samples were embedded in polymethylmethacrylate (PMMA) (Technovit 9100, Heraeus-Kulzer, Germany) for visual histological analysis. Two sets of sequential sections were stained with Giemsa and alizarin red. Fifty *μ*m thick sequential sections were cut on a saw microtome (SP1600, Leica, Germany). Sections were analysed and digitized by light microscopy (Axioscope A1, Axiovision LE release 4.8.2, Carl Zeiss, Germany). Digitized images were merged, to create overview images, using Photoshop CS3 (Adobe Systems, USA).

### 2.6. Statistics

Statistical analysis was performed in SPSS (version 21, IBM, USA) using a Mann-Whitney *U* test for 2-tailed significance. *P* values below 0.05 were regarded as significant. Graphs were made in GraphPad Prism 5 (GraphPad, USA).

## 3. Results

### 3.1. Preparation of *β*-TCP and Optimization of the Paste Composition

**Figure 1:**
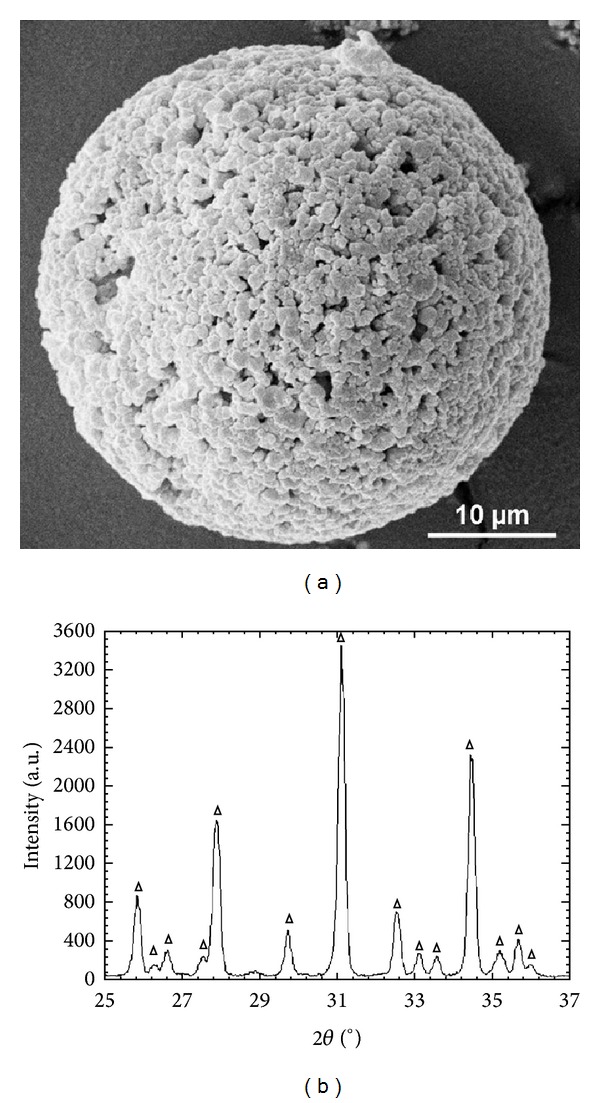
*β*-TCP data. (a) SEM micrograph of a spray dried *β*-TCP granule. (b) XRD diagram of the spray dried *β*-TCP granule, which only shows the presence of *β*-TCP (triangle).

The XRD measurements of the heat treated hydroxyapatite confirmed that a phase pure *β*-TCP was obtained. The specific surface area of the material was 1.84 m^2^/g. The d90 value of the grain size was 16.3 *μ*m. After the milling process, the surface area was extended to 3.38 m^2^/g. The d90 value of the grain size was reduced to 5.0 *μ*m. The spray-dried granules had a compact and spherical shape (Figure 1(a)). The presence of *β*-TCP was confirmed by XRD analysis (Figure 1(b)).

The cement composition used to plot the eventual scaffolds was established after sequential optimisation steps. The addition of trisodium citrate, PEG, and water resulted in stable extrudable paste which allowed drying at room temperature without compromising the integrity of the plotted scaffold. The density of the final cement composition was 1.66 ± 0.05 g/cm^3^.

**Figure 2:**
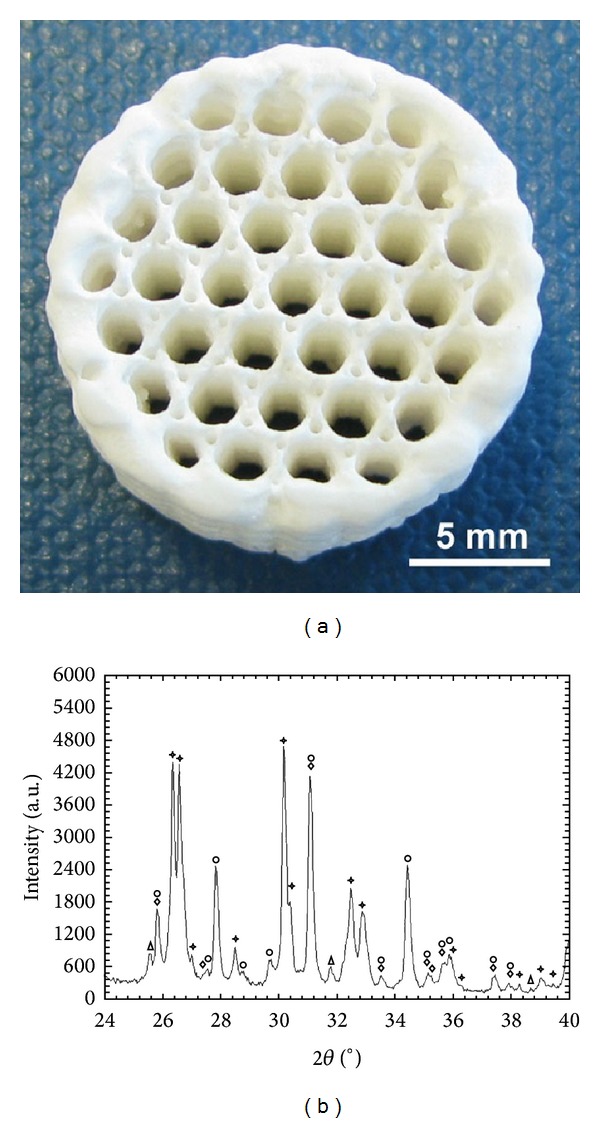
Scaffold material evaluation. (a) A cylindrical scaffold synthesized by extrusion freeform fabrication using *β*-TCP, PEG 400, PEG 10000, trisodium citrate, and water as cement material. The scaffold was hardened with phosphoric acid. (b) XRD diagram of an extruded scaffold. Four chemical phases, whitlockite (diamond), monetite (asterisk), sodium calcium phosphate (circle), and hydroxyapatite (triangle), were detected.

The manufactured scaffolds had a compact edge and exhibited an open porosity in the centre of the cylinder (Figure 2(a)). The compression strength of the plotted scaffold lattice was 1.2 ± 0.6 MPa. The XRD analysis indicated besides the presence of the original phase *β*-TCP (Ca_3_(PO_4_)_2_) (mineral name: whitlockite) also the presence of hydroxyapatite (Ca_5_(PO_4_)_3_OH), monetite (CaH (PO_4_)) and sodium calcium phosphate (Ca_10_Na (PO_4_)_7_) (Figure 2(b)). Hardening of the scaffold with phosphoric acid resulted in a solid calcium phosphate composition. The washing step with water and the subsequent drying resulted in a cured composition consisting of monetite and hydroxyapatite. The addition of PMMA to the cement did not influence the manufacturing process of the scaffold nor its mechanical stability.

Furthermore the scaffold structural integrity was not affected by incubation in culture medium for a period of 4 weeks under standard culture conditions.

### 3.2. Cell Cytocompatibility Tests

**Figure 3:**
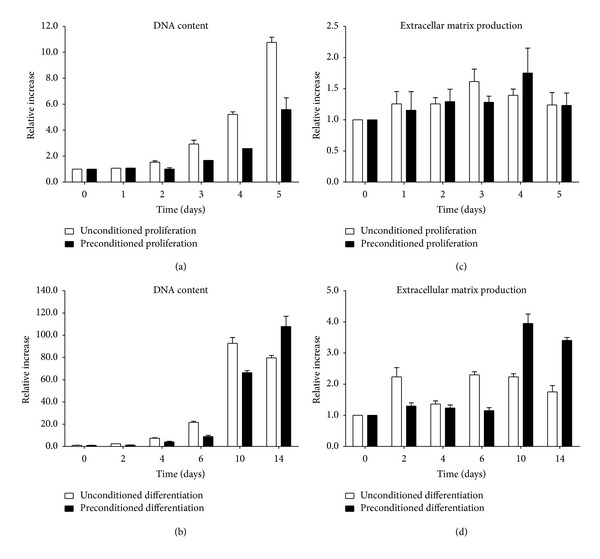
DNA content (crystal violet staining) and extracellular matrix production levels (alcian blue staining) of ATDC5 cells in preconditioned proliferation and differentiation medium. The absorbance of the extracted crystal violet and alcian blue was used to calculate the DNA content and extracellular matrix production relative to the absorbance value at day 0. (a) DNA content during proliferation conditions. (b) DNA content during differentiation conditions. (c) Extracellular matrix production during proliferation conditions. (d) Extracellular matrix production during differentiation conditions.

Since ATDC5 cells are widely used for the assessment of endochondral mechanisms *in vitro* [25–27], these cells were used for the evaluation of *in vitro* cytocompatibility of the developed scaffold material. Crystal violet staining confirmed that the ATDC5 cells proliferated and differentiated in both unconditioned and preconditioned culture medium (Figures 3(a) and 3(b)). The proliferation rate in the preconditioned samples was on average one day slower compared to the unconditioned control samples. This could suggests either that the developed scaffold material marginally influences cell proliferation in its direct vicinity or that the scaffold material exhibits a toxic effect on the ATDC5 cells, although no cell death was detected. However, during 14 days of differentiation the preconditioned samples eventually outperform the unconditioned samples. This is also confirmed by alcian blue staining (Figures 3(c) and 3(d)), where the preconditioned samples generate more extracellular matrix after 14 days of differentiation as compared to the unconditioned samples and the cells grown under proliferation conditions.

### 3.3. *In Vivo* Evaluation of Plotted Scaffolds

To address the overall osteocompatibility *in vivo*, the 16 mm wide, 10 mm thick, 3D plotted scaffolds (Figure 2(a)) were implanted in a critical size defect in the iliac wing of sheep. The implantation of the plotted scaffold material into the sheep iliac wing did not result in any detected form of inflammation or pathological reaction like necrosis during the experimental follow-up. This was also indicated by *in vitro* cytocompatibility tests. All animals were euthanized 12 weeks after implantation, after which the area surrounding the defects was excised from the iliac wing, by a core drill, and subsequently fixated in 70% ethanol.

**Figure 4:**
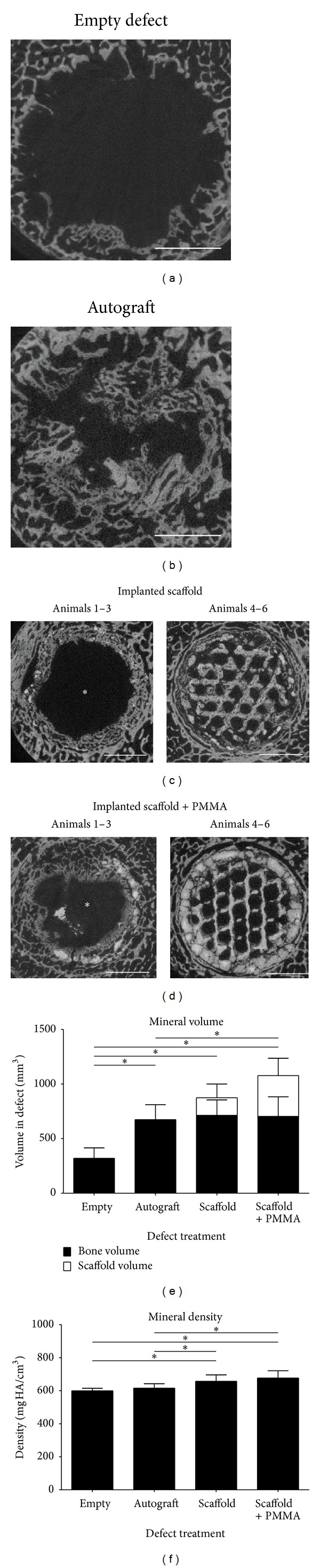
MicroCT analysis and quantification. (a) Representative microCT image of an empty defect. (b) Representative microCT image of an autograft control defect. (c) Representative microCT images of implanted *β*-TCP scaffolds. Asterisk indicates scaffold resorption in animals 1, 2, and 3. (d) Representative microCT images of implanted *β*-TCP/PMMA scaffolds. Asterisk indicates scaffold resorption in animals 1, 2, and 3. (e) Quantification of the mineralized tissue volume in the defect region. (f) Quantification of the mineral density of the mineralized tissue in the defect region. Size bars indicate 5 mm, the error bars indicate the standard deviation, the asterisk indicates *P* < 0.05.

Quantitative microCT imaging revealed that the empty defects showed limited remodelling in the defect area (Figure 4(a)), while the autograft shows the presence of bony tissue in the defect (Figure 4(b)). The implanted *β*-TCP scaffold material showed evident bone remodelling in the defect with evident scaffold resorption in about 50% of the animals (Figure 4(c), asterisk). This effect was also noted in the *β*-TCP/PMMA scaffold group (Figure 4(d), asterisk), although it appeared that this effect was animal dependent (Table 1). Quantification of the mineral volume in the defect region indicated that the autograft treated defects had significantly more bone remodelling compared to the empty defects (Figure 4(e)) (*P* = 0.004). Although there was significantly more mineral volume present in the autograft filled defect, there was no significant difference between the density of the mineral tissue in the empty and the autograft defect (Figure 4(f)) (*P* = 0.200). Both scaffold treated defects had significantly more bone remodelling in the defect region compared to the empty defect (Figure 4(e)) (*P* = 0.004). Furthermore only the *β*-TCP/PMMA scaffold had a significantly higher mineral volume present in the defect region compared to the autograft treatment (Figure 4(e)) (*P* = 0.037); the mineral volume of the *β*-TCP scaffold showed a trend towards significance (Figure 4(e)) (*P* = 0.078). The total density of all mineral structures present in the defect (mineralized tissue including the scaffold material) was significantly higher compared to both the empty defect and the autograft control group (for the *β*-TCP scaffold *P* = 0.004 and 0.037, respectively, and for the *β*-TCP/PMMA scaffold *P* = 0.004 and 0.016, respectively, Figure 4(f)). This suggests that the scaffold itself functions as a supporting structure for the remodelled bony structure and contributes to the density of the mineral composition in the defect.

**Figure 5:**
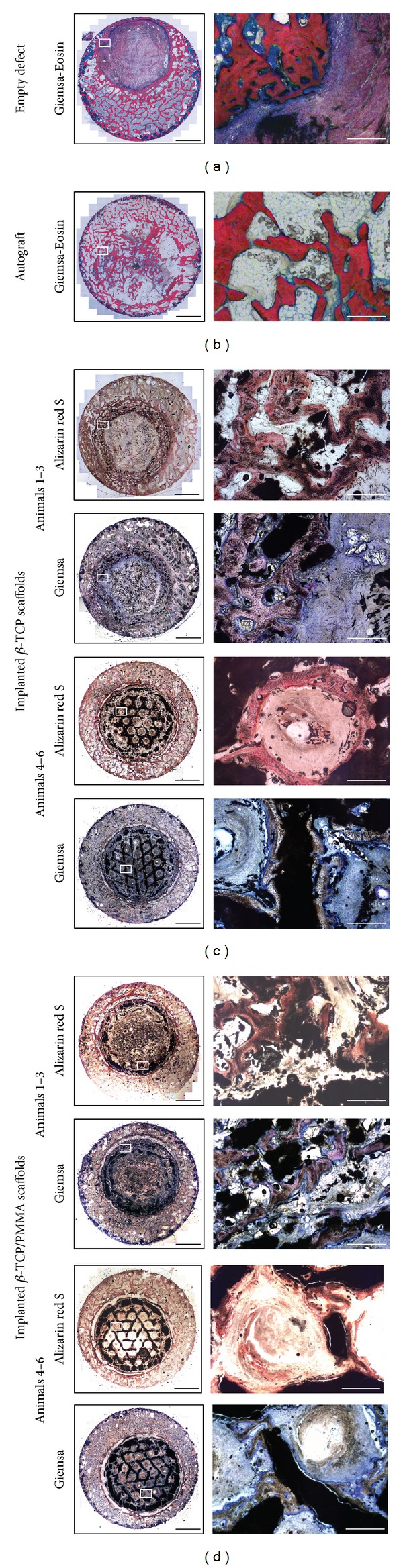
Histological sections of the control and the experimental groups. (a) Giemsa-eosin stain of an empty defect. (b) Giemsa-eosin stain of an autograft control. (c) Giemsa and Alizarin red stained sections of the implanted *β*-TCP scaffolds. Scaffold resorption was detected in animals 1, 2, and 3, while remaining present in animals 4, 5, and 6. (d) Giemsa and Alizarin red stained sections of the implanted *β*-TCP/PMMA scaffolds. Scaffold resorption was detected in animals 1, 2, and 3, while remaining present in animals 4, 5, and 6. Black bars represent 5 mm and white bars represent 500 *μ*m.

Visual analysis of the histological sections of the empty defects (Figure 5(a)) showed limited bone growth into the defect area, while the autograft control (Figure 5(b)) showed the presence of bony tissue in the defect. The implantation of the *β*-TCP and *β*-TCP/PMMA scaffold (Figures 5(c) and 5(d)) supported bone deposition into the defect; however resorption of the scaffold was noted in 50% of the animals after 12 weeks. Still bone remodelling can be noted around the leftover scaffold particles. This resulted in a remaining defect in 50% of the animals, yet smaller compared to the originally created defect and the remaining defect in the empty defect control group. Still this kind of scaffold resorption was found to be an animal dependent, bilateral effect (Table 1). Importantly, in the other animals, the scaffold structure was still present in its original form, showing bone deposition in the honeycomb-like structure of the scaffold, indicating the potential of the scaffold material to serve as a supporting structure during bone remodelling in critical size defects.

## 4. Discussion

The present study shows that generation of calcium phosphate scaffolds at room temperature by extrusion freeform fabrication using suspensions with high solid matter content strongly depends on the properties of the ceramic paste. Furthermore, scaffolds extruded at room temperature generally have a higher density, compared to scaffolds established by extrusion combined with freeze drying [28, 29]. It was found that filter pressing, thread thickness, and thread stability during extrusion and after drying are influenced by the combination of steric and electrostatic additives in the composition. Only the right combination of long and short chain polymers with an electrostatic additive provides the necessary properties for good extrudable paste, including drying at room temperature and maintaining structural integrity after drying. This suggests that the composition of the mixture is essential for its extrusion characteristics, indicating an essential balance between the individual compounds. Other studies proved that the extrusion freeform fabrication of calcium phosphate scaffolds is possible, although a thermal heating process was included [30, 31]. The material in our study showed similar capabilities regarding the production process without the necessity of a high temperature after processing step.

The new chemical phases that occurred after the hardening process are a result of the reaction with phosphoric acid. Phosphoric acid reacts with *β*-TCP by forming new calcium phosphate phases [11]. Due to a high precipitation kinetic, dicalcium phosphate dihydrate (DCPD) forms at the beginning of the process [32]. As a result of the acidic pH finally dicalcium phosphate (DCP, monetite) is being formed from the DCPD [11]. The increase in pH by rinsing the scaffolds with water after hardening results in the formation of HA. During this precipitation process sodium, from the trisodium citrate, is incorporated into the apatite phase, which often occurs at physiological pH [33, 34]. At the end, *β*-TCP is still one of the main compositions in addition to monetite, hydroxyapatite, and sodium calcium phosphate.

The final strength of the scaffold lattice (0.9 ± 0.3 MPa) was slightly beneath that of natural spongiosa [35] but still within the same range as other calcium phosphate scaffolds fabricated either at low temperature or by solid free form fabrication [36, 37].

Furthermore, this study indicated a good cytocompatibility of the new cement formulation *in vitro*. The extension to *in vivo* implantation of the scaffold material into the iliac wing of sheep has shown that the material itself is suitable for the production of 3D scaffolds. The plotted scaffolds also possess relevant properties for clinical applications, for example, material handling properties and stability during implantation.

The histological data of the implanted scaffolds showed that the scaffolds function as a supporting framework for the mineralized tissue, since all bone tissue mineralisation within the scaffold matrix is in the direct contact with the scaffold surface.

Interestingly, the scaffolds degraded completely and bilaterally in some animals, whereas in others the scaffolds remained present. Although in aspect of age, sex, and weight there was no significant difference between the animals, the reason of this different degradation rate remains unclear and needs further investigation. Nevertheless, the scaffold implanted group performed significantly better in terms of the amount of mineral volume and density in the defect compared to the empty defect control group. These first findings are promising; however more animal data with more time points to evaluate scaffold degradation and bone formation is needed including the comparison with existing bone substitutes.

Furthermore, the production procedure of these cements allows the potential incorporation of a release system. A drug-releasing polymer or microbeads could be implemented into these scaffolds, since the described scaffold material does not require a sintering step [37, 38]. The hardening of the scaffolds is performed by a short immersion in phosphoric acid, similar to other researchers using 3D powder printing [36]. Still the acid could hamper the effectiveness of incorporated drugs or proteins. However, other researchers already showed that drug release is possible even when a weak phosphoric acid is directly incorporated in the mixing solution [39]. Our data show that loading of these scaffolds with PMMA granules does not negatively influence the scaffold on either physical or *in vivo* biological properties.

## 5. Conclusion

In the present pilot study, we developed and evaluated novel calcium phosphate compositions for the production of scaffolds, for critical size bone defects, at room temperature. The *in vitro* and *in vivo* evaluation methods were used to assess to cytocompatibility and bone conductive properties of the material, respectively.

Our data clearly showed the potential of these cements in the field of orthopaedic or maxillofacial surgery, for treatment of critical size defects or serving as a bone substitute. Still the design of the internal scaffold structure should be adjusted to allow the application in loadbearing situations. For now this scaffold material could serve as a basis for a novel scaffold platform which allows incorporation of a secondary component like PMMA for drug-releasing applications.

## Figures and Tables

**Table 1:** Scaffold presence after 12 weeks based on microCT and histology.

Sheep #	Left iliac wing	Right iliac wing	Outcome
1	*β*-TCP scaffold	*β*-TCP/PMMA scaffold	Bilateral scaffold resorption
2	*β*-TCP/PMMA scaffold	*β*-TCP scaffold	Bilateral scaffold resorption
3	*β*-TCP scaffold	*β*-TCP/PMMA scaffold	Bilateral scaffold resorption
4	*β*-TCP/PMMA scaffold	*β*-TCP scaffold	Scaffold remains present bilaterally
5	*β*-TCP scaffold	*β*-TCP/PMMA scaffold	Scaffold remains present bilaterally
6	*β*-TCP/PMMA scaffold	*β*-TCP scaffold	Scaffold remains present bilaterally
